# Trabecular and cortical bone mineral density in human vertebrae: comparison of frozen and thawed States with qCT

**DOI:** 10.1007/s00068-025-03056-6

**Published:** 2026-01-13

**Authors:** Titus Kühlein, Adrian Cavalcanti Kußmaul, Sandy Walter, Jan Wulf, Richard Zaccaria, Paul Reidler, Wolfgang Böcker, Christoph Linhart

**Affiliations:** 1 Department of Orthopaedics and Trauma Surgery, Musculoskeletal University Center Munich (MUM), LMU University Hospital, LMU Munich, Marchioninistr. 15, 81377 Munich, Germany; 2 Department of Radiology, University Hospital, LMU Munich, Munich, Germany

**Keywords:** Quantitative computed tomography (qCT), Volumetric bone mineral density (vBMD), Freeze-Thaw cycle, Trabecular and cortical bone, Bone preservation, Forensic osteology, Bone quality assessment, Osteoporosis

## Abstract

**Purpose:**

Bone mineral density (BMD) is a key determinant of skeletal strength, relevant in many disciplines. Freezing is widely used to preserve cadaveric bone, but its effect on BMD remains controversal. This study assessed whether a vBMD is altered in frozen versus thawed state of human vertebral bodies measured by quantitative computed tomography (qCT).

**Materials and methods:**

Thirty-three human vertebrae (14 C7, 9 Th1, 10 Th2) were stored at − 20 °C. Trabecular and cortical vBMD were measured in the frozen state (− 15 to − 20 °C) using qCT with a calibration phantom. After thawing overnight at room temperature, identical scans were performed. Paired t-tests and Pearson correlation coefficients compared frozen and thawed values.

**Results:**

One trabecular specimen was excluded as an outlier. Mean trabecular volometeic bone mineral density (vBMD) was 154.68 mg Ca-HA/ml (frozen) vs. 148.93 mg Ca-HA/ml (thawed), mean difference − 5.74 mg Ca-HA/ml (*p* = 0.164). Cortical vBMD was 219.56 mg Ca-HA/ml (frozen) vs. 219.90 mg Ca-HA/ml (thawed), mean difference 0.34 mg Ca-HA/ml (*p* = 0.948). Correlations between frozen and thawed values were strong (*r* = 0.89 trabecular; *r* = 0.94 cortical).

**Conclusion:**

A single freeze–thaw cycle at − 20 °C does not significantly alter cortical or trabecular vBMD in human vertebral bodies measured by qCT. These results support the measurement of BMD of frozen specimens, minimizing handling and avoiding unnecessary thawing in research and forensic settings.

## Introduction

 Bone mineral density (BMD) is a critical determinant of skeletal strength and plays a fundamental role in a number of disciplines, including orthopedic research, forensic analysis and tissue engineering. Trabecular bone, distinguished by its porous structure, exhibits heightened sensitivity to environmental and preservation conditions, which can impact its mechanical properties and mineral density [1]. Consequently, the storage and handling of bone specimens represent essential considerations in both research and clinical applications. Freezing is a widely used method to preserve biological tissues, but its effect on bone density and structural properties remains controversial.

In theory, ice is slightly less dense and offers marginally less X-ray absorption than liquid water for the same mass and has shown differences in X-ray absorption spectra. Furthermore freezing has proven to alter the microstructure of bone tissue [3, 16]. In order to determine the overall effect of these various influencing factors on bone density measurement, empirical testing is carried out on human bone specimens in this study.

Prior studies rely on different storage temperatures. Some studies have conducted tests under storage conditions of −20 degrees [2], while others have used − 80 degrees [3], which is the established standard for some tissue banks. However, it is currently assumed that the different temperatures have comparable effects on the bone [4].

Measuring BMD is recommended as part of quality checks on bone specimens before performing tests on human cadavers, but this is not currently conducted by default [5, 6]. To reduce the amount of freezing and thawing cycles measurements of bone density are usually performed directly before testing [5]. Unfortunately, this is not always easy to achieve due to logistical issues. Furthermore, biomechanical tests with paired specimens have shown that bone density should be taken into account when pairing [5]. Accordingly, the bone density of all study specimens must be measured before performing tests on the first one. This raises the question of whether measuring bone density in the frozen state yields comparable results to those obtained in the thawed state, thereby preventing the need for thawing and refreezing. The present study investigates this question using frozen human vertebral bodies.

Earlier studies have been conducted on animal cadaver specimens, employing rabbit or pig bones, leading to ambiguous results [7–9]. For example, Amanda et al. examined the bone density of seven pig specimens over time as they were frozen. They found that the fresh initial scan and final frozen scan were significantly different (*p* < 0.001), whereas the final thawed BMD scans did not differ from the initial fresh scans [9]. On the other hand, Trudel et al. found no difference in bone density between frozen (0.31 ± 0.08 g/cm^2^ 95%CI from 0.30 to 0.32 g/cm^2^) and thawed calcanei (0.31 ± 0.08 g/cm^2^ 95% CI from 0.30 to 0.32 g/cm^2^; paired t-test *p* > 0.01) in their study of 56 rabbit calcanei [8].

In addition, there are several studies that have used human bones [2, 3, 8, 14]. Here, too, the results were controversial: Wähnert et al. analyzed the bone mineral density in 19 frozen and thawed human femora using DXA, finding significantly higher (by 1.4%) density in the frozen 0.827 g/cm^2^ ± 0.15 state compared to the thawed one 0.816 g/cm^2^ ± 0.15; *p* = 0.006 [10]. On the other hand, Trudel et al. did not find any significant difference in their comparative study on 102 human calcanei (*p* > 0.01) [8].

The majority of studies used Dual-Energy X-Ray (DXA) to determine bone density [8, 10–12, 14]. Its analysis is limited to two-dimensional bone structure measurement due to the recording technique and its limitations should be noted. Accordingly, areal bone density (g/cm²) is measured rather than volumetric density (mg/cm³), making it susceptible to bone size. While DXA remains clinically practical, its inability to distinguish between bone compartments limits its application in biomechanical and microstructural research. Important influencing factors for DXA measurements include anatomical changes, such as degenerative changes, and malpositioning of specimens in relation to the X-ray source during repeat testing. Additionally, body fat percentage and its distribution can also alter the result.

Especially the influence of the surrounding soft tissue leads to challenges in the comparable measurement of frozen and thawed specimens. Various techniques have been established in this regard. In some cases, attempts have been made to simulate soft tissue by embedding the specimens in rice [8], and in others by embedding them in water [10]. This problem is irrelevant for qCT, as it creates a three-dimensional image of the object, thereby enabling measurements to be taken of selected areas. Additionally, it may avoid the overestimation of BMD by DXA associated with spinal degeneration, other sclerosis lesions, such as bone islands and abdominal aortic calcification [13].

Furthermore, qCT can distinguish between different structures within the bone itself. On one hand there is the cortical bone. Consisting of a compact microstructure and containing hardly any water, changes in density between frozen and thawed bones are unlikely. On the other hand there is the trabecular bone, containing much more water, and a porous structure. To get further insights, both types of bone structure were measured and analyzed separately.

This study addresses the hypothesis that freezing bones causes a relevant change in bone density and accordingly, bone mineral density measurements should only be performed on thawed bones to generate accurate values. To the best of our knowledge, this is the first study to analyze human cadaver vertebras using qCT to obtain data about the volumetric bone density whilst permitting a differentiation between corticalis and the spongiosa. We thereby aim to provide further valuable insights for forensic pathology, orthopedic surgery, and bone tissue preservation.

## Materials and methods

### Specimen collection and storage

In this in vitro biomechanical study 33 human vertebrae were used, consisting of fourteen C7, nine Th1 and ten Th2. These vertebral segments were selected in order to make a statement regarding the less frequently tested cervical and thoracic spine and at the same time to ensure high homogeneity regarding the shape and size of the corpora. After extraction, all specimens were immediately frozen at −20 °C in sealed plastic bags to prevent freeze-drying and desiccation. Their use in this study was approved in advance by the ethical committee of the LMU Munich (EA 20–1128).

### Quantitative computed tomography (qCT) analysis

Volumetric bone mineral density (vBMD) was measured using quantitative computed tomography (qCT) (Somatom Definition Edge^®^, Siemens Healthengineers, Munich, Germany). The scanning procedure was performed by a trained radiologist according to a standardized protocol.

**Fig. 1:**
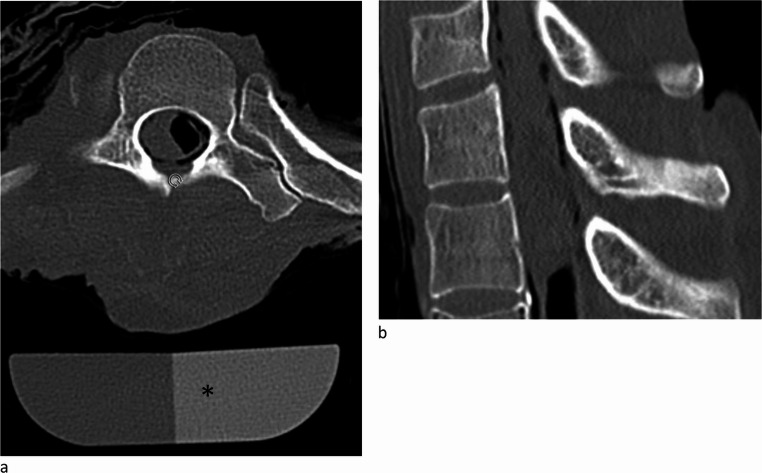
CT-scans showing a vertebra in axial (1a) and sagittal (1b) view. *indicates the phantom that is used as reference to correctly determine the bone density

To ensure accurate and reliable measurements, a calibration phantom (a reference material of known density) was placed under or beside the sample during each scan (Fig. 1a). This calibration process allowed standardization of the bone mineral density (BMD) measurements, expressed in mg Ca-HA/ml.

The acquired CT images were processed using dedicated qCT software (Syngo Osteo CT^®^, Siemens Healthengineers, Munich, Germany). This software automatically segmented and differentiated the trabecular and cortical bone compartments for separate analysis.

### Experimental procedure and statistical analysis

All specimens were analyzed in the frozen state at approximately − 15 °C to −20 °C. After initial scanning, the vertebrae were thawed overnight at room temperature. The following day, the specimens underwent a second qCT scan using the same standardized imaging protocol to assess potential changes in bone density due to the thawing process. Statistical analyses were performed using MB SPSS Statistics^®^ version 29 (Armonk, NY, USA).

With the intention of performing a paired t-test, we checked all available data for extreme values (more than 3 times the interquartile range away from both quartiles) and performed a Shapiro Wilk test to prove normal distribution. The strength of the linear relationship between frozen and thawed BMD values was assessed using the Pearson correlation coefficient. Descriptive statistics, including mean values, standard deviations, and 95% confidence intervals, were also calculated.

## Results

### Trabecular bone density

**Fig. 2:**
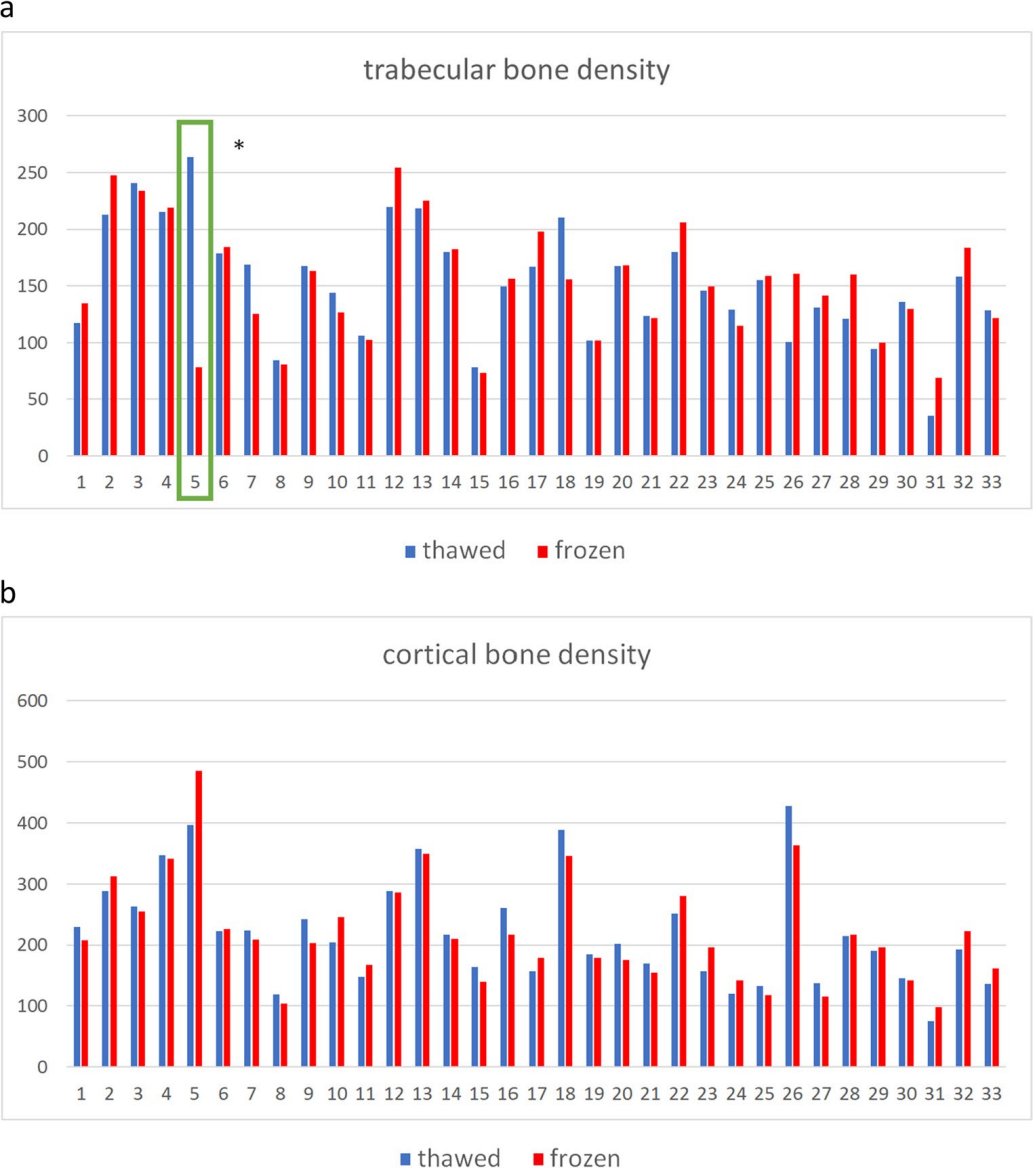
**a** Calculated bone density of the spongiosa in Ca-HA/ml of all tested specimens in the frozen and thawed state. * Indicates extreme values (more than 3 times the interquartile range away from both quartiles) and was therefore exclude. It is likely that the determination of the trabecular structure in the thawed status included partly cortical bone and is therefore a lot higher than the compared frozen status. The numbers correlate to the following vertebras: 1–15 C7, 16–24 Th1, 25–33 Th2. **b**: calculated bone density of the cortical bone in Ca-HA/ml of all tested specimens in the frozen and thawed state. The numbers correlate to the following vertebras: 1–15 C7, 16–24 Th1, 25–33 Th2

The test for extreme values led to the exclusion of sample number six C7 (Fig. 2a). The mean trabecular bone mineral density (BMD) was 154.68 mg Ca-HA/ml in the frozen state and 148.93 mg Ca-HA/ml in the thawed state (Table 1). The standard deviations were 49.53 and 47.60, respectively. The mean difference between the two states was − 5.74 mg Ca-HA/ml, with 95% confidence intervals ranging from − 13.96 to 2.48. After demonstrating a normal distribution using the Shapiro-Wilk test (*p* = 0.67), a paired two-sample t-test was performed. This produced a t-statistic of −1.42 with 31 degrees of freedom (df). The p-value for a two-sided test was 0.164, indicating that the observed difference was not statistically significant (*p* > 0.05) (Table 1). The Pearson correlation coefficient between frozen and thawed trabecular BMD was 0.89, indicating a strong correlation between the two conditions.

**Table 1:** Descriptive statisctics and two-sample t-test with dependent samples (paired comparison test)

	Bone density thawed trabecular in mg Ca-HA/ml	Bone density frozen trabecular in mg Ca-HA/ml
Mean	148.93	154.68
Standard deviation	47.60	49.53
Observations	32.00	32.00
Mean difference	−5.74	
95% confidence intervals (lower/upper limit)	−13.96/2.48	
T	−1.42	
Correlation	0.89	
Degrees of freedom (df)	31	
Significance (p) one-sided/two-sided	0.82/0.164	
	Bone density thawed cortical in mg Ca-HA/ml	Bone density frozen cortical in mg Ca-HA/ml
Mean	219.90	219.56
Standard deviation	87.14	87.16
Observations	33.00	33.00
Mean difference	0.34	
95% confidence intervals (lower/upper limit)	−10.30/10.99	
T	0.66	
Correlation	0.941	
Degrees of freedom (df)	32	
Significance (p) one-sided/two-sided	0.474/0.948	

### Cortical bone density

No extreme values were found in the cortical bone density group (Fig. 2b). The mean BMD in the frozen state was 219.56 mg Ca-HA/ml, compared to 219.90 mg Ca-HA/ml in the thawed state (Table 1). The standard deviations were 87.16 and 87.14, respectively. The mean difference was 0.34 mg Ca-HA/ml, with 95% confidence intervals ranging from − 10.30 to 10.99. After demonstrating a normal distribution using the Shapiro-Wilk test (*p* = 0.375), a paired two-sample t-test was performed. This produced a t-statistic of 0.66 with 32 degrees of freedom (df). The p-value for a two-sided test was 0.948, confirming that there was no statistically significant difference (*p* > 0.05) (Table 1). The Pearson correlation coefficient for cortical BMD was 0.94, indicating a very strong correlation between frozen and thawed states.

## Discussion

This study aimed to evaluate the effect of freezing on volumetric bone mineral density (vBMD) in human vertebral specimens using quantitative computed tomography (qCT), which allows compartment-specific analysis of cortical and trabecular bone. Our findings demonstrate that even though mean vBMD values differed slightly between frozen and thawed states, these differences did not reach statistical significance for either trabecular (*p* = 0.164) or cortical bone (*p* = 0.948). Consequently these findings do not support the hypothesis that there are notable differences in bone mineral density between frozen and thawed cadaver specimens.

This study builds upon prior investigations. Wähnert et al. examined 19 fresh-frozen human femora in their study, both in the frozen and thawed state [10]. The frozen state was at −20 degrees Celsius. They found a decrease in BMD after thawing in 14 of the 19 femora. The measured total BMD was 1.4% higher in the frozen state, which was significant (*p* = 0.006). The technique used here was DXA. Accordingly, the authors of this study recommend measuring bone density only on thawed specimens [10].

Trudel et al. came to different conclusions with a significantly larger study size. They also investigated the difference in bone density between frozen and thawed specimens. They conducted a study on 56 calcanei from 28 female New Zealand white rabbits and 102 calcanei from 51 human donors. The measurements were taken using DXA at −13 °C and + 20 °C. No differences in bone density were found in either sub-study (rabbit study 0.00 ± 0.02 g/cm², *p* < 0.01; human specimens 0.00 ± 0.04 g/cm², *p* < 0.01). Accordingly, these authors did not recommend preferential testing in the thawed state [8].

Another study that addresses this subject is that of Amanda et al. [9]. In two sub-studies, the influence of freezing on the bone density of fetal pigs was examined in short-term and long-term tests. In the testing of three specimens, in fresh condition and after five days of freezing, using DXA, no significant difference was found. However, in weekly measurements of four fetal pigs over a period of 20 weeks, they found a decrease in BMD. This was significant (*p* < 0.001). Nevertheless, the final thawed BMD scans did not differ from the initial fresh scans [9]. It should be noted that fetal bones have a different ratio of cortical and trabecular bone than adult bones and their transferability is therefore limited.

The inconsistencies in results of previous studies may be attributable not only to differences in measurement techniques and anatomical locations but also to the heterogeneous behavior of cortical versus trabecular bone under freeze-thaw conditions.

Although our results did not show a statistically significant effect, the trabecular bone showed a slightly greater mean difference in bone density (−5.74 mg Ca-HA/ml) and a lower p-value compared to the cortical bone (0.34 mg Ca-HA/ml). This trend—alongside the relatively wide confidence intervals and moderate sample size—raises the possibility that a true effect may exist but remained undetected in this study due to insufficient statistical power. Increasing the sample size in future studies may clarify whether these subtle differences reflect genuine biological variability or are within the range of expected measurement noise.

On the other hand, the strong correlation between frozen and thawed measurements suggests that, despite minor differences, the preservation state does not meaningfully impair the reliability of vBMD assessments using qCT. This is particularly relevant for forensic medicine, orthopedic research and the handling of bio banked skeletal tissues, where freeze-thaw cycles are often unavoidable.

There are some limitations of this study that should be noted. Firstly, the bones were not tested directly after being harvested, but rather after being thawed. This means that any changes to the tissue caused by the freezing process may also have been altered in the thawed test samples. However, since many experiments with bone specimens, especially biomechanical tests, are almost always carried out in a thawed state after a certain freezing phase, this study provides realistic insights.

As another limitation it should be mentioned that no reliability tests regarding the qCT measurements were performed. A high variation in repetitive testing may therefore cover up minor differences. However, the possibility of a systemic bias does not affect the relationship between frozen and thawed testing, since both were tested equally. Additionally, qCT and the radiological analysis protocol used in this study are well established and offer high reliability.

Furthermore, it should be mentioned that the exact sampling period of the tested specimens is not known. Consequently, even though the specimens were measured at the same time, no uniform freezing duration can be determined. However, the time of storage seem to have little effect on the bone density [15].

Finally, this study only addresses the determination of bone density as a parameter for assessing bone quality. Many studies have attempted to analyze the effect of freezing on microarchitecture and determine other bone parameters [3, 16]. Taillebot et al., for example, conducted a study on femoral heads and analyzed possible changes in microarchitecture after 6 weeks at −80 degrees compared to freshly harvested samples. These were scanned using an X-ray microcomputed tomography scanner (µCT) to obtain the microarchitectural parameters, including the bone volume fraction, the mean trabecular thickness, the trabecular separation, the degree of anisotropy, and the connectivity density. There was no statistically significant difference between the fresh and frozen groups for any of the parameters measured [3]. Nevertheless, densitometry remains an important tool with high everyday relevance due to its wide availability and deeper insights to microarchitecture remain reserved for specific questions.

## Conclusion

This study provides novel data on the effect of a single freeze–thaw cycle on human vertebral vBMD measured by qCT. Statistical analysis revealed no significant differences in trabecular bone between frozen 154.68 ± 49.53 mg Ca-HA/ml and thawed 148.93 ± 47.60 mg Ca-HA/ml conditions. Also, regarding the cortical bone no significant difference between frozen 219.56 ± 87.16 mg Ca-HA/ml and thawed 219.90 mg ± 87.14 Ca-HA/ml state was found. While trabecular BMD showed a slight post-thaw decrease, p-values confirmed the variation was not statistically meaningful (*p* = 0.164). Accordingly, we see no evidence in this study to support the hypothesis that bone freezing has a relevant influence on bone density. Based on these results, frozen cadaver specimens can be confidently used for bone density measurements, avoiding unnecessary thawing and refreezing as well as simplifying handling of specimens.

## Data Availability

No datasets were generated or analysed during the current study.
